# A brief introduction to health economics

**DOI:** 10.1007/s00167-019-05372-y

**Published:** 2019-02-07

**Authors:** Norman Waugh, Hema Mistry

**Affiliations:** Division of Health Sciences, Warwick Medical School, University of Warwick, Gibbet Hill Campus, Coventry, CV4 7AL UK

**Keywords:** Cost-effectiveness, Quality-adjusted life year, Health economics

## Abstract

To convince policy-makers or funders of health care of the value of orthopaedic interventions, we need to consider value for money (cost-effectiveness), as well as clinical effectiveness. This article provides an introduction to health economics to set the scene for papers on the use of allografts in the knee.

## Introduction

The four papers that follow summarise a health technology assessment (HTA) commissioned by ESSKA to inform development of a guideline on the use of allografts in the knee. The aim was to assess the cost-effectiveness of allografts for various indications in the knee. This article provides a brief introduction to health economics to help readers with the terminology and methods in the subsequent papers.

## Review

HTA research addresses the following questions:

- Does it work?
- At what cost?
- Is it worth it, compared to other possible uses of funds?

This is done to help policy-makers or commissioners of healthcare with a fourth question: should they provide it?

Underlying all of this is the hard reality that funds are always scarce and that publicly-funded health services are never able to fund every treatment that might do some good for some people. Therefore, choices have to be made about what to fund and what not to fund. Do policy makers fund osteochondral allografts or cancer drugs? The true cost of funding one treatment is what economists call “opportunity cost”—some other intervention cannot be funded.

The benefits of health care range from symptoms relieved, to lives prolonged (years of life gained). We capture the various benefits through the common currency known as the quality-adjusted life year (QALY), which captures both length of life and quality of life in one single index measure. One QALY = 1 year of perfect life. This makes it possible to compare treatments that prolong lives, with treatments that do not affect length of life, but improve the quality of life. Some treatments affect both the length and the quality of life. For example, some cancer drugs will prolong life, even if only for a few months, but may have unpleasant side-effects that reduce quality of life. Therefore, the QALYs gained will be less than life years gained.

If someone with a painful knee has quality of life reduced by 20%—a “utility” reduction of 0.2 on a scale of 0–1.0, where 0 is dead and 1.0 is perfect health—then, each year of life is worth only 0.8 QALYs. If an intervention removes or reduces the knee pain that increases quality of life and leads to an increase in QALYs. For example, clinical success and failure 5 years after autologous chondrocyte implantation (ACI) compared with microfracture were evaluated. Success after ACI led to an increase in utility to 0.817, compared to a baseline utility score of 0.654, which equates to 0.815 QALYs over 5 years [5].

If our patient has knee pain sufficient to reduce quality of life by 20%, for 10 years, that is a loss of 2.0 QALYs. If an intervention restores utility to normal, we gain 2 QALYs. Based on the cost of the intervention and all other associated costs and savings, we can then estimate the cost per QALY.

Health care costs should include all costs and savings. Costs include:

- The costs of assessment and treatment.
- The costs of any adverse effects.
- The costs of rehabilitation or other follow-up.

Treatment costs need to include all the costs of providing it. Therefore, the costs of an orthopaedic operation include not just the theatre and ward staff costs, but all the costs required to support that operation, including medical records, reception staff, overheads (i.e., heating, lighting, and maintenance), finance staff and personnel department costs, and so on. All these costs have to be recovered by hospitals in the charges they make for each case. Private hospitals may add on a bit to pay shareholders.

Health care savings include:

- The costs of conservative care avoided, such as analgesics and physiotherapy, or aids to mobility. These may be considerable over years. Some patients may incur pain that requires powerful pain killers, and addiction can occur.
- The costs of joint replacement if that can be avoided or postponed. Note that postponing a first knee arthroplasty may mean that a more expensive revision arthroplasty may never be required.

One issue that we have to take into account is discounting [9]. This involves adjusting future costs and benefits to current values. It is based on the principle that £1000 or $1000 is valued more highly if received now, than in the future. The UK Government applies a 3.5% discount rate to future costs and benefits. Therefore, £1000 now is valued at £965 in 1 year time. This has implications for interventions that lead to future costs avoided. For example, if a knee replacement costs £6000 today, the discounted cost is £4201 at 10 years and £3516 at 15 years. Therefore, if the intervention now leads to knee replacement avoided in 10 years, the savings are only assessed to be £4201. However, discounting can also support interventions that postpone knee replacements—if an intervention now postpones future knee replacement from 10 to 15 years that equates to a saving of £4201–£3516 = £658. In cost-effectiveness analysis, the future saving reduces the current cost of the intervention by £658.

Other countries apply different discount rates, and sometimes, the rates differ for costs and outcomes, for example, 6% for costs and 1.5% for benefits.

Discounting has been hotly debated, with some arguing that costs should be discounted but benefits not, but in practice, decision-making processes are based on discount rates set by governments. Discounting may disadvantage interventions that prevent future problems, such as intensive diabetes treatment to prevent renal failure in 20 year time.

The cost per QALY that is considered affordable various amongst countries. In the UK, the National Institute for Health and Care Excellence (NICE) regards anything under £20,000 per QALY as acceptable, and will also consider costs per QALY in the £20,000–£30,000, depending on the strength of evidence [4]. In some circumstances, NICE will accept a much higher cost per QALY, but so far, none of those have been in orthopaedics. Therefore, for our patient with the 20% reduction in quality of life for 10 years, NICE would consider that an investment of £40,000–£60,000 would be justified to restore full quality of life, even without considering possible future savings.

There are also costs and savings for patients. These may include monetary costs such as loss of income from time of work. Economic analysis may note such costs, but they are often not included in cost-effectiveness analysis, as only costs incurred by the health care service are assessed. However, non-monetary costs such as inability to participate in normal activities can be picked up by quality-of-life scores.

A common problem in orthopaedic studies is that many of the outcome measures used such as Knee injury and Osteoarthritis Outcome Score (KOOS) [10] or the Western Ontario Meniscal Evaluation Tool (WOMET) [6] do not convert to a generic utility score such as the EQ-5D [3] or SF-6D [2]. We need the “common currency” of utility scores that can measure quality of life across a range of conditions or disease, to derive QALYs. If we have to decide whether to spend £500,000 on orthopaedics or cancer, we need to know how many QALYs we can get from each of those. We need the utility measure, so that we can compare the health gains from improving quality of life by orthopaedic interventions, with life years gained from cancer care (while adjusting for quality-of-life reductions from side-effects).

Some clinical scores such as Western Ontario and McMaster Universities Osteoarthritis Index (WOMAC) [7] have been mapped to the EQ-5D to generate utility scores [1, 11, 12], but it would be much better if future orthopaedic studies included a generic utility measure such as the EQ-5D. This would make it easier to do cost-effectiveness analysis in orthopaedics and might strengthen orthopaedic bids in the competition for limited health care funds.

However, we do need condition or disease-specific outcomes measures as well, since they may be more sensitive to changes that matter to patients. Not every change can be quantified in the EQ-5D. For example, an individual may score well on EQ-5D despite restriction of activities that were previously enjoyed, but not regarded by policy makers as an essential part of activities of daily living.

Not all linear changes on clinical scores will have the same effects on quality of life. For example, in macular eye disease, there may be a series of equal deteriorations in visual acuity, but the step that stops people driving has more impact than most.

Another issue for cost-effectiveness analysis is that trials, and other studies needs to provide data on effect size. It is not enough to say “Treatment A was better than treatment B, *p* < 0.01”—we need to know how much better treatment A was than treatment B. Some statistically significant differences may be clinically and economically insignificant. Sometimes, a difference, which meets the minimally significant clinical difference threshold, may be economically insignificant.

Not all effects, good or bad, can be precisely quantified. However, they can be taken account of by applying a bit of judgment in the appraisal process. One author (NW) of this article was involved in a NICE appraisal of insulin pumps, which can make management of diabetes easier in children. One effect is that parental quality of life may be improved. Parents, especially mothers, may need to be less “on call” for problems at school and this can affect their employment. NICE noted that.

Uncertainties are common because of lack of data. For example, many studies of knee cartilage repair procedures do not have long enough follow-up data to assess impact on knee replacement, or the numbers with long follow-up may be small. In cost-effectiveness analysis, uncertainties are addressed by “sensitivity analysis”. A “base case” is created using the likeliest values and then the effect of varying the modelling assumptions is tested by applying higher or lower values instead. This can be useful for identifying the factors with the greatest effect, and those with little effect.

Cost-effectiveness of a procedure may vary amongst patients, according to patient characteristics. For example, a previous health technology assessment report found that the cost-effectiveness of autologous chondrocyte implantation was better amongst patients who had not had any previous repair attempts that damaged the subchondral bone [8]. The cost per QALY also increased according to Kellgren–Lawrence grade of osteoarthritic change.

The reality of savings needs to be considered. A new version of an operation may take 10 min less operating time, but unless that means that more operations can be done in a theatre session, or that staff hours can be reduced, there will be no real savings. Similarly, if the new procedure reduces length of hospital stay, there will be no savings unless staffing levels are reduced. Indeed, there may be an increase in costs if the capacity saved is used for additional cases. This may be good for patients, but a problem for the hospital finance directors.

Unlike drugs, devices or procedures evolve over time, and the only long-term results may be from superseded versions. The base–case analysis could assume that newer versions are as good, but then, we can explore various sensitivity analyses, assuming that the latest version is 10% better or 20% better, to see what that does to the cost per QALY.

## Conclusion

HTA aims to aid decision-making by those who make decisions on allocation of funds, but can also inform clinicians and support bids for funding of future services. As will be seen in an accompanying article, osteochondral allografting for people with osteochondral defects has a remarkably low cost per QALY, so would be regarded as highly cost-effective by bodies such as NICE.

## Abbreviations

HTA: Health technology assessment
QALY: Quality-adjusted life year
ACI: Autologous chondrocyte implantation
EQ-5D: The EuroQol measure of quality of life

## References

[1] Barton GR, Sach TH, Jenkinson C, Avery AJ, Doherty M, Muir KR (2008). Do estimates of cost-utility based on the EQ-5D differ from those based on the mapping of utility scores?. Health Qual Life Outc.

[2] Brazier J, Roberts J, Deverill M (2002). The estimation of a preference-based measure of health from the SF-36. J Health Econ.

[3] Brooks R (1996). EuroQol: the current state of play. Health Policy.

[4] Claxton K, Martin S, Soares M, Rice N, Spackman E, Hinde S (2015). Methods for the estimation of the National Institute for Health and Care Excellence cost-effectiveness threshold. Health Technol Assess.

[5] Gerlier L, Lamotte M, Wille M, Kreuz PC, Vanlauwe J, Dubois D (2010). The cost utility of autologous chondrocytes implantation using ChondroCelect(R) in symptomatic knee cartilage lesions in Belgium. Pharmacoeconomics.

[6] Kirkley A, Griffin S, Whelan D (2007). The development and validation of a quality of life-measurement tool for patients with meniscal pathology: the Western Ontario Meniscal Evaluation Tool (WOMET). Clin J Sport Med.

[7] McConnell S, Kolopack P, Davis AM (2001). The Western Ontario and McMaster Universities Osteoarthritis Index (WOMAC): a review of its utility and measurement properties. Arthritis Rheum.

[8] Mistry H, Connock M, Pink J, Shyangdan D, Clar C, Royle P (2017). Autologous chondrocyte implantation in the knee: systematic review and economic evaluation. Health Technol Assess.

[9] National Institute for Health and Care Excellence (2013) Guide to the methods of technology appraisal 2013: process and methods [PMG9]. https://www.nice.org.uk/process/pmg9/chapter/foreword. Accessed 4 Dec 2018 27905712

[10] Roos H, Lauren M, Adalberth T, Roos EM, Jonsson K, Lohmander LS (1998). Knee osteoarthritis after meniscectomy: prevalence of radiographic changes after twenty-one years, compared with matched controls. Arthritis Rheum.

[11] Wailoo A, Hernandez Alava M, Escobar Martinez A (2014). Modelling the relationship between the WOMAC Osteoarthritis Index and EQ-5D. Health Qual Life Outc.

[12] Xie F, Pullenayegum EM, Li SC, Hopkins R, Thumboo J, Lo NN (2010). Use of a disease-specific instrument in economic evaluations: mapping WOMAC onto the EQ-5D utility index. Value Health.

